# Age difference of patients with and without invasive aspergillosis: a systematic review and meta-analysis

**DOI:** 10.1186/s12879-024-09109-2

**Published:** 2024-02-19

**Authors:** Elena Shekhova, Fabián Salazar, Alessandra Da Silva Dantas, Tanmoy Chakraborty, Eva L. Wooding, P. Lewis White, Adilia Warris

**Affiliations:** 1 Medical Research Council Centre for Medical Mycology, Geoffrey Pope Building, University of Exeter, University of Exeter, Stocker Road, Exeter, EX4 4QD UK; 2 School of Dental Sciences, Newcastle University, Framlington Place, Newcastle upon Tyne, NE2 4BW UK; 3 Royal Devon and Exeter Hospital, Exeter, EX2 5DW UK; 4 Public Health Wales Microbiology Cardiff, Cardiff University, UHW, Cardiff, UK; 5 Centre for Trials Research, Division of Infection and Immunity, Cardiff University, UHW, Cardiff, UK

**Keywords:** Aspergillosis, Age, Systematic review, Meta-analysis

## Abstract

**Background:**

Invasive Aspergillosis (IA) is a life-threatening fungal disease with significant mortality rates. Timely diagnosis and treatment greatly enhance patient outcomes. This study aimed to explore the association between patient age and the development of IA, as well as the potential implications for risk stratification strategies.

**Methods:**

We searched National Center for Biotechnology Information (NCBI) databases for publications until October 2023 containing age characteristics of patients with and without IA. A random-effects model with the application of inverse-variance weighting was used to pool reported estimates from each study, and meta-regression and subgroup analyses were utilized to assess sources of heterogeneity.

**Results:**

A systematic review was conducted, resulting in the inclusion of 55 retrospective observational studies with a total of 13,983 patients. Meta-analysis revealed that, on average, patients with IA were approximately two and a half years older (95% Confidence Interval [CI] 1.84–3.31 years; I^2^ = 26.1%) than those without the disease (*p* < 0.0001). No significant moderators could explain the observed heterogeneity in age difference. However, subgroup analysis revealed that age differences were more pronounced within particular patient groups compared to others. For example, patients with and without IA who had primary severe lung infections exhibited a greater difference in mean age than other patient cohorts.

**Conclusions:**

Further research, such as individual patient data meta-analysis, is necessary to better understand the potential relationship between increasing age and the likelihood of IA. Improved risk stratification strategies based on patient age could potentially enhance the early detection and treatment of IA, ultimately improving patient outcomes.

**Supplementary Information:**

The online version contains supplementary material available at 10.1186/s12879-024-09109-2.

## Introduction

*Aspergillus* spp. are among the most common causes of fungal infections in humans and can cause a variety of diseases in immunocompromised individuals and those with chronic lung disorders [1]. Invasive aspergillosis (IA) is estimated to affect roughly 200,000 people every year, with an overall mortality rate of 50%, reaching 100% if misdiagnosed [2]. The timely diagnosis and treatment of IA might be challenging as the available fungal diagnostic tests lack sensitivity. Thus, accurate knowledge of risk factors that predispose individuals to IA is essential for pre-empting disease, initiating early treatment, and ultimately improving survival [3].

Several patient-related risk factors are well-known to be associated with IA. Prolonged neutropenia is a key risk factor and often plays a role in patients with hematologic malignancies and those receiving chemotherapy and/or bone marrow transplantation. Solid organ transplantation and the use of immunosuppressive therapy, as well as prolonged use of corticosteroids have been recognized as risk factors for developing IA [3]. Interestingly, in the last 20 years, IA has been described in patients lacking classical risk factors. Emerging at-risk groups now include non-neutropenic patients such as those being critically ill, those with severe viral pneumonias, and those who receive novel biological therapies targeting immune-signaling pathways [4]. The identification of specific host factors predisposing individuals to IA is essential for early recognition of the development of IA, and can also be used to design specific management strategies (e.g., antifungal prophylaxis).

Expanding our understanding of the link between aging and susceptibility to infectious diseases is imperative as the number of elderly adults (aged over 60 years old) grows every year and has estimated to reach 2.1 billion by 2050 (World Health Organization; updated 2021 Sep 10). Currently, it is well established that aging affects the immune system and lung function, making older individuals more susceptible to respiratory infections like influenza and COVID-19 [5]. However, it remains unclear whether clinicians need to consider patients’ age when managing patients at risk of IA. Multiple clinical studies reported age as an independent risk factor for IA, while others could not find any association between older age and risks for IA. To investigate this further, a systematic review and meta-analysis were performed with the primary aim of identifying whether individuals with IA are older or younger than those without infection. Our ultimate objective was to guide future research and improve clinical estimates on the possibility of IA.

## Materials and methods

The systematic review and subsequent meta-analysis were conducted according to the PRISMA guidelines (Table S1). Prior to the completion of the systematic search by the first reviewer, the study was preregistered on the preregistration service of the Open Science Framework (OSF, Registration DOI: 10.17605/OSF.IO/HFMP7).

### Systematic literature search and inclusion of relevant studies

The literature search was performed to retrieve available studies in which the age of patients who were infected with IA was reported separately to patients who did not acquire this infection. We used the PICO framework to specify the eligibility criteria for the studies [6]. The diagnosis of IA in patients with underlying conditions was used as a selection criterion. IA was defined as proven, probable, or putative, as classified by individual studies [7, 8]. No specific interventions were investigated. For the comparison, the age of patients who acquired IA was compared to the age of patients who were not diagnosed with the disease. The control group included either all patients without IA in a cohort or randomly selected from the pool of patients without the disease in case-control studies. The reported mean or median age in both groups was used as an outcome.

R programming software and the RISmed package were utilized to search for reports in the National Center for Biotechnology Information (NCBI) databases. The query was created using the keywords: invasive aspergillosis risk factors. Age was not included in the keywords for the reason of not missing studies where age was reported but was not identified as a major risk factor. The search was performed with the following combination of terms: invasive [All Fields] AND (“aspergillosis” [MeSH Terms] OR “aspergillosis” [All Fields]) AND (“risk factors” [MeSH Terms] OR (“risk” [All Fields] AND “factors” [All Fields]) OR “risk factors” [All Fields]). The resulting studies were filtered with the following constraints: written in English and published before 2023/10/01. The appearance of duplicates was assessed.

One reviewer (ES) and a team of three reviewers (AD, FS, TC) independently evaluated the titles and abstracts for relevance using the automated abstract screener available with the metagear [9]. Records were excluded if they were presented as single case reports, non-clinical laboratory research, reviews (including systematic reviews), studies focusing on various invasive fungal infections, preliminary results from trials, clinical guidelines, therapeutic drug monitoring, non-human studies, clinical investigations focusing only on pediatric patients, studies on chronic aspergillosis, studies describing solely clinical characteristics of IA, publications without abstracts. Selected relevant full-text records were further evaluated. Discrepancies were settled by consensus.

### Data extraction

Two authors (ES, EW) conducted data extraction from included studies using spreadsheets, which were reviewed for accuracy and comprehensiveness. The following variables were recorded: year of publication, years of data collection, patient’s primary disease, administration of antifungal prophylaxis (even when administrated only to a portion of patients), study type (cohort or case-control), and the requirement for intensive care management. When age was expressed as a median, the authors of such studies were contacted to provide the mean age and standard deviation. If this was not provided, the median age and the first and third quartiles were recorded to estimate the approximate sample mean and standard deviation.

### Statistical analysis

Statistical analysis was performed using the RStudio software Version 1.4.1717. The following packages were utilized: dplyr, meta, metafor, dmetar, ggplot2, patchwork, flextable [10–16]. The dataset and the script of the analysis were uploaded at https://osf.io/hfmp7. The meta package was used to calculate and pool effects estimates. The effect size was calculated as a mean difference. To approximate means from the sample sizes, medians, and first and interquartile range, we used a method described by Luo et al. [17]. Approximate standard deviations were calculated from the same statistics using the method proposed by Shi et al. [18]. A random-effects model was applied to pool the results of each study with inverse-variance weighting. We used a restricted maximum-likelihood estimator for between-study variance tau^2^ [19]. Q-profile method for confidence interval of tau^2^ and tau [20]. The method proposed by Hartung and Knapp was applied to adjust test statistics and confidence intervals [21]. Outliers were defined as studies, in which the confidence interval did not overlap with the confidence interval of the pool effects analysis and were removed [22, 23]. Meta-analysis data was presented without outliers, effect size, prediction interval, and I^2^ heterogeneity before and after the removal of the outliers presented in Supplemental Table S2.

The dataset was further examined to evaluate the presence of possible influential studies that had a substantial impact on the overall result of the analysis. The baujat plots [24] did not detect any studies which would overly contribute to the heterogeneity (Fig. S1). The leave-one-out method was further utilized to estimate how omitting each study would affect the overall effect size and I^2^ heterogeneity (Fig. S2). This analysis showed that the exclusion of any of the studies from the analysis would not substantially alter heterogeneity and the effect size, confirming the absence of influential cases in the final dataset. For meta-regression and subgroup analyses, levels of moderator (study-level) variables were incorporated based on a pre-planned analysis of specific parameters of included studies [23]. Moderator variables were included in a random-effects model, which yielded a mixed-effects meta-regression model. The resulting value of I^2^ in this model reveals the amount of residual heterogeneity in the true effects, while R^2^ denotes the amount of heterogeneity that might be explained by the inclusion of a moderator in the model.

### Data quality measures

Both publication bias and study quality bias were assessed via evaluation of small study bias [25, 26]. This was done by visually examining a funnel plot (Fig. S3) and implementing Egger’s regression test [25]. The evaluation did not indicate a small study bias as Eggers’ test did not show the presence of funnel plot asymmetry with *p-*value of 0.9727. In addition, a contour-enhanced funnel plot, which includes key areas of statistical significance (*p* = 0.1, *p* = 0.05, *p* = 0.01) (Fig. S4), was created to identify the risk of publication bias [27].

### Newcastle-Ottawa quality assessment of studies

The quality and risk of bias in observational studies included in the meta-analysis was assessed using the Newcastle-Ottawa Scale tool [28]. The studies we evaluated based on three key components: the selection of study groups (involving representativeness and unbiased control selection), the comparability of groups (including the control for additional factors), and the rigor of outcome assessment (encompassing the ascertainment of diagnosis and the duration of follow-up). Notably, since none of the included studies were explicitly designed to examine age-related characteristics of patients, control for significant additional factors was lacking. Consequently, none of the studies qualified for a score in the comparability component, resulting in an overall assessment of either fair or poor quality according to the Agency for Healthcare Research and Quality (Table S3).

## Results

**Fig. 1:**
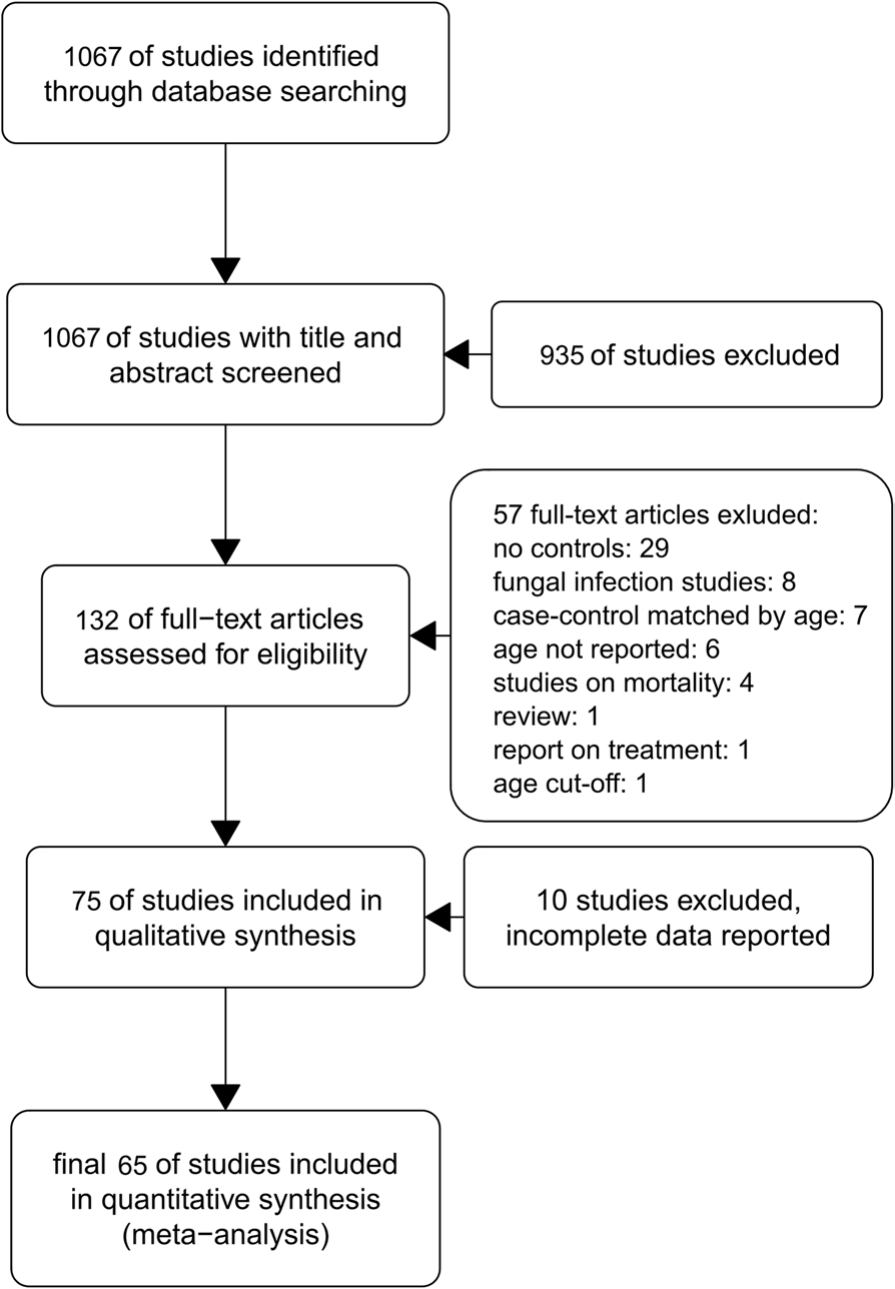
A flow diagram illustrating the process of selecting studies for inclusion in the analyses

 Of the 1067 studies screened, 65 were included in the meta-analysis (Fig. 1) [29–93]. Studies were either a retrospective cohort (*n* = 46) or case-control (*n* = 19) design and were published before October, 2023. The main characteristics of selected studies are described in Table 1, while the quality assessment is presented in Table S3.

**Table 1:** Overview of included studies

Publication	Underlying condition	ICU	Prophylaxis^a^	Sample size	Study design	IA type^b^	Start year	End year
Feys et al., 2023 [92]	COVID-19	YES	NO	335	cohort	mixed	2020	2022
Hurt et al., 2023 [86]	COVID-19	YES	NO	266	cohort	probable	2020	2021
Dai et al., 2023 [82]	Hematologic disease	NO	NO	189	cohort	mixed	2011	2021
Dubler et al., 2023 [93]	Critically ill	YES	YES	121	cohort	putative	2015	2019
Grootveld et al., 2023 [83]	COVID-19	YES	NO	793	cohort	mixed	2020	2021
Song et al., 2023 [80]	Hematologic disease	NO	NO	67	cohort	probable	2021	2022
Waldeck et al., 2023 [81]	Influenza	YES	NO	158	cohort	mixed	2017	2020
Lee et al., 2022 [90]	COVID-19	NO	NO	228	cohort	mixed	2020	2021
Kim et al., 2022 [72]	COVID-19	YES	NO	187	cohort	mixed	2020	2021
Er et al., 2022 [74]	COVID-19	YES	NO	213	cohort	mixed	2020	2021
Calderon-Parra et al., 2022 [76]	COVID-19	YES	NO	84	case-control	mixed	2020	2021
Bentvelsen et al., 2022 [88]	COVID-19	YES	NO	123	case-control	mixed	2020	2020
de Almeida et al., 2022 [78]	COVID-19	YES	NO	56	case-control	mixed	2020	2021
Xu et al., 2021 [77]	COVID-19	YES	NO	335	cohort	mixed	2019	2020
Chao et al., 2021 [84]	Influenza	YES	NO	90	cohort	mixed	2016	2018
Apostolopoulou et al., 2021 [91]	Organ transplantation	NO	NO	224	cohort	mixed	2011	2017
Katada et al., 2022 [89]	Organ transplantation	NO	YES	120	case-control	mixed	2011	2016
Janssen et al., 2021 [87]	COVID-19	YES	NO	279	cohort	mixed	2020	2020
Prattes et al., 2022 [85]	COVID-19	YES	NO	592	cohort	mixed	2020	2021
Le Pavec et al., 2021 [75]	Organ transplantation	NO	NO	191	cohort	mixed	2013	2017
Gu et al., 2021 [73]	COPD	NO	NO	616	cohort	mixed	2012	2017
Lahmer et al., 2021 [79]	COVID-19	YES	NO	32	case-control	putative	2020	2020
Xu et al., 2021 [77]	Hematologic disease	NO	NO	91	cohort	mixed	2016	2019
Razazi et al., 2020 [44]	ARDS	YES	NO	172	cohort	putative	2009	2020
Delliere et al., 2021 [41]	COVID-19	YES	NO	108	cohort	probable	2020	2020
Chauvet et al., 2020 [39]	COVID-19	YES	NO	46	cohort	putative	2020	2020
Bellelli et al., 2020 [42]	Influenza	NO	NO	77	cohort	NR	2018	2019
Chen et al., 2020 [37]	Pneumonia	NO	NO	693	cohort	mixed	2018	2018
Waldeck et al., 2020 [36]	Influenza	YES	NO	81	cohort	NR	2017	2018
Seok et al., 2020 [46]	Organ transplantation	NO	NO	78	case-control	probable	1995	2015
Zou et al., 2020 [62]	Pneumonia	NO	NO	335	cohort	probable	2013	2018
Sharma et al., 2020 [56]	Influenza	NO	NR	477,556	cohort	NR	2005	2014
Lahmer et al., 2019 [35]	Liver disease	YES	NO	84	cohort	probable	NR	NR
Tejerina et al., 2019 [43]	Critically ill	YES	NO	878	cohort	proven	1991	2016
Napolioni et al., 2019 [31]	Hematologic disease	NO	NO	352	cohort	NR	NR	NR
Bitterman et al., 2019 [48]	Hematologic disease	NO	NO	107	cohort	proven	2015	2018
Levesque et al., 2019 [29]	Liver disease	YES	NO	208	case-control	mixed	2005	2015
Huang et al., 2019 [57]	Influenza	YES	NO	109	case-control	mixed	2017	2018
Herrera et al., 2019 [52]	Organ transplantation	NO	NO	23	case-control	mixed	2016	2016
Cook et al., 2018 [60]	Organ transplantation	NO	NO	69	case-control	mixed	1986	2015
Rodriguez-Goncer et al., 2018 [51]	Critically ill	YES	NO	125	cohort	putative	2012	2016
Kaya et al., 2017 [47]	Hematologic disease	NO	YES	152	case-control	mixed	2010	2012
Zhang et al., 2018 [59]	Liver disease	NO	NO	1077	cohort	NR	2011	2016
White et al., 2017 [33]	Hematologic disease	NO	NO	274	cohort	proven	2005	2009
Lopez-Medrano et al., 2018 [50]	Organ transplantation	NO	NO	122	case-control	mixed	2000	2013
Lopez-Medrano et al., 2016 [65]	Organ transplantation	NO	NO	102	case-control	mixed	2000	2013
Nagao et al., 2016 [53]	Liver disease	NO	YES	30	case-control	mixed	2007	2013
Heylen et al., 2015 [45]	Organ transplantation	NO	NO	123	case-control	mixed	1995	2013
Kurosaki et al., 2014 [40]	Pneumonia	NO	NO	539	cohort	proven	2006	2012
Gustot et al., 2014 [68]	Liver disease	YES	NO	94	cohort	mixed	2006	2012
Luong et al., 2014 [38]	Organ transplantation	YES	YES	93	cohort	mixed	2006	2010
Chen et al., 2013 [49]	Liver disease	YES	NO	87	case-control	mixed	2008	2012
Schwarzinger et al., 2013 [70]	Hematologic disease	NR	YES	185	cohort	mixed	2003	2006
Wauters et al., 2012 [54]	Pneumonia	YES	NO	40	cohort	mixed	2009	2011
Xu et al., 2012 [61]	COPD	NO	NO	90	case-control	probable	2006	2009
Michallet et al., 2012 [34]	Hematologic disease	NO	NO	261	cohort	mixed	2004	2007
Michallet et al., 2011 [30]	Hematologic disease	NO	YES	117	case-control	probable	2006	2008
Mikulska et al., 2009 [32]	Hematologic disease	NO	YES	304	cohort	mixed	1999	2006
Kontoyiannis et al., 2007 [71]	Hematologic disease	NR	YES	66	case-control	NR	2002	2004
Garnacho-Montero et al., 2005 [55]	Critically ill	YES	NO	756	cohort	mixed	1998	1999
Muhlemann et al., 2005 [69]	Hematologic disease	NO	YES	142	cohort	proven	1995	1999
Fukuda et al., 2004 [64]	Hematologic disease	NO	YES	2319	cohort	mixed	1992	2001
Thursky et al., 2004 [67]	Hematologic disease	NR	YES	206	cohort	mixed	1991	1998
Munoz et al., 2004 [63]	Organ transplantation	NO	YES	278	cohort	mixed	1998	2002
Hahn et al., 2002 [66]	Hematologic disease	NR	YES	35	cohort	mixed	1992	1992

*ICU *intensive care unit, *ARDS *acute respiratory distress syndrome, *NR *not reported, *COPD *chronic obstructive pulmonary disease

^a^Prophylaxis was stated as YES even when administrated only by a portion of patients, the following agents were used for antifungal prophylaxis: [93]: fluconazole/caspofungin; [89]: itraconazole; [47]: posaconazole; [53]: fluconazole/micafungin; [38]: amphotericin B/voriconazole/itraconazole; [30]: posaconazole; [32]: fluconazole, fluconazole/amphotericin B, amphotericin B, voriconazole; [64]: fluconazole/itraconazole; [67]: fluconazole/itraconazole; [63]: itraconazole; [70]: amphotericin B/fluconazole/itraconazole; [71]: NA; [66]: itraconazole; [58]: posaconazole; [69]: fluconazole

^b^In the data collection process, the IA type was recorded as mixed when the age of patients was not specifically categorized or stratified based on the IA classification

**Fig. 2:**
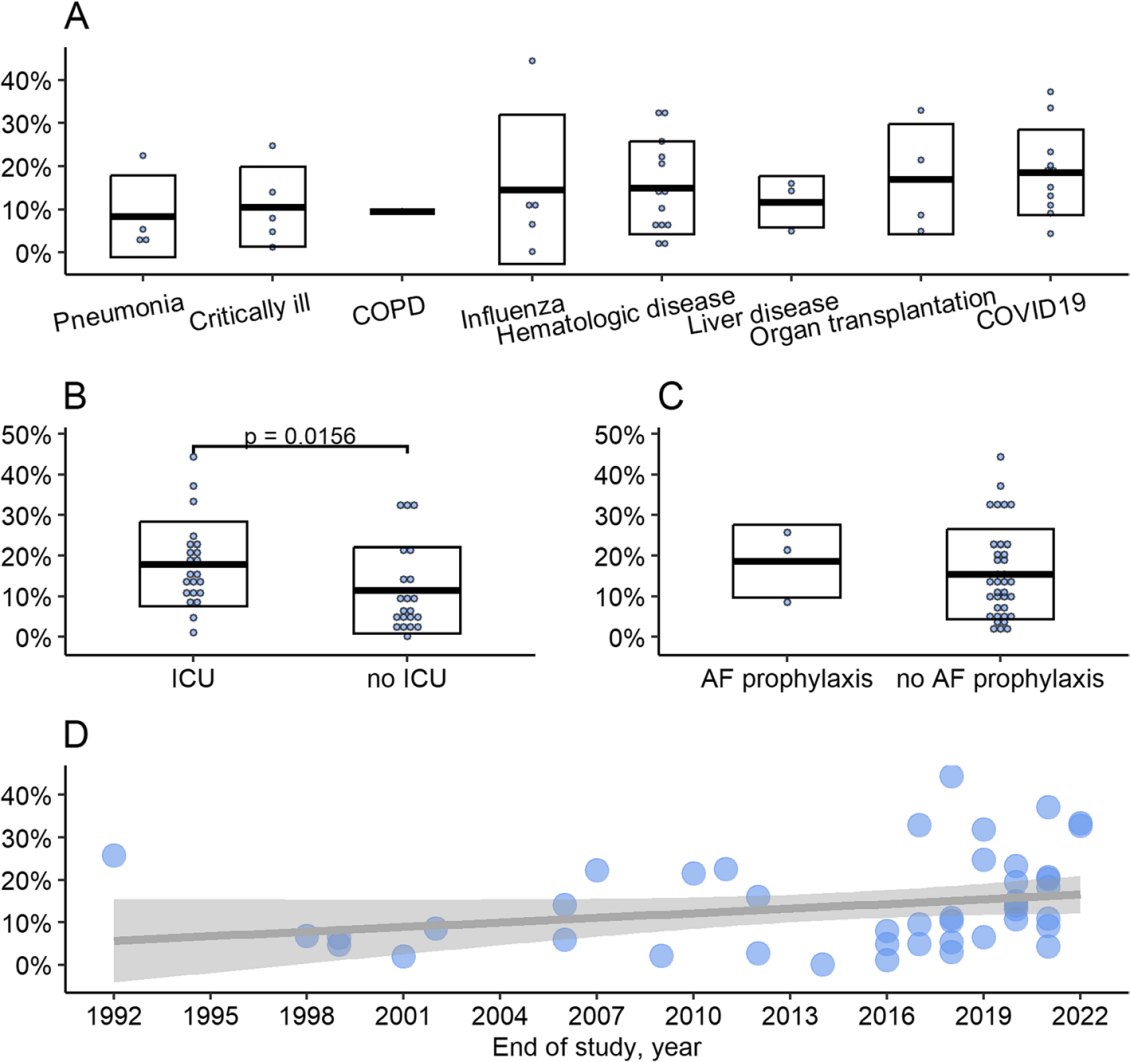
Variations in incidence of IA: patient types, treatments, and trends over time

 As a part of the characterization of studies, differences in the incidence of IA across different groups of patients and over time were evaluated using cohort studies (Table 1) involving 492,192 patients. This analysis showed that the incidence of IA did not significantly vary in patients with different underlying conditions as identified by using the Kruskal–Wallis test followed by the Dunn’s test indicating *p* > 0.05 (Fig. 2A). Incidence of IA was significantly higher in ICU patients compared to those who did not require admission to ICU (*p* = 0.0156, two-samples unpaired test Wilcoxon test) (Fig. 2B). Also, in our data set, the incidence of IA did not differ in the patient cohort that receives mold-active antifungal prophylaxis compared with those not receiving prophylaxis at all (*p* = 0.497, two-samples unpaired test Wilcoxon test) (Fig. 2C). Finally, we tested whether the incidence of IA has changed over time. The robust linear regression model indicated a possible relationship between disease incidence and the year when the data collection was concluded (Fig. 2D). The model’s result suggested a possible increase in incidence by 0.36% per year, but this increase was not statistically significant, as indicated by the *p*-value of 0.12.

Prior to performing the final meta-analysis, we carried out a sensitivity analysis, ten studies were indicated as outliers and removed [31, 37, 46, 47, 56, 70, 73, 81, 84, 89]. In total, 55 studies involving 13,983 patients (12,045 patients without and 1,938 with IA) were included in the final meta-analysis.

**Fig. 3:**
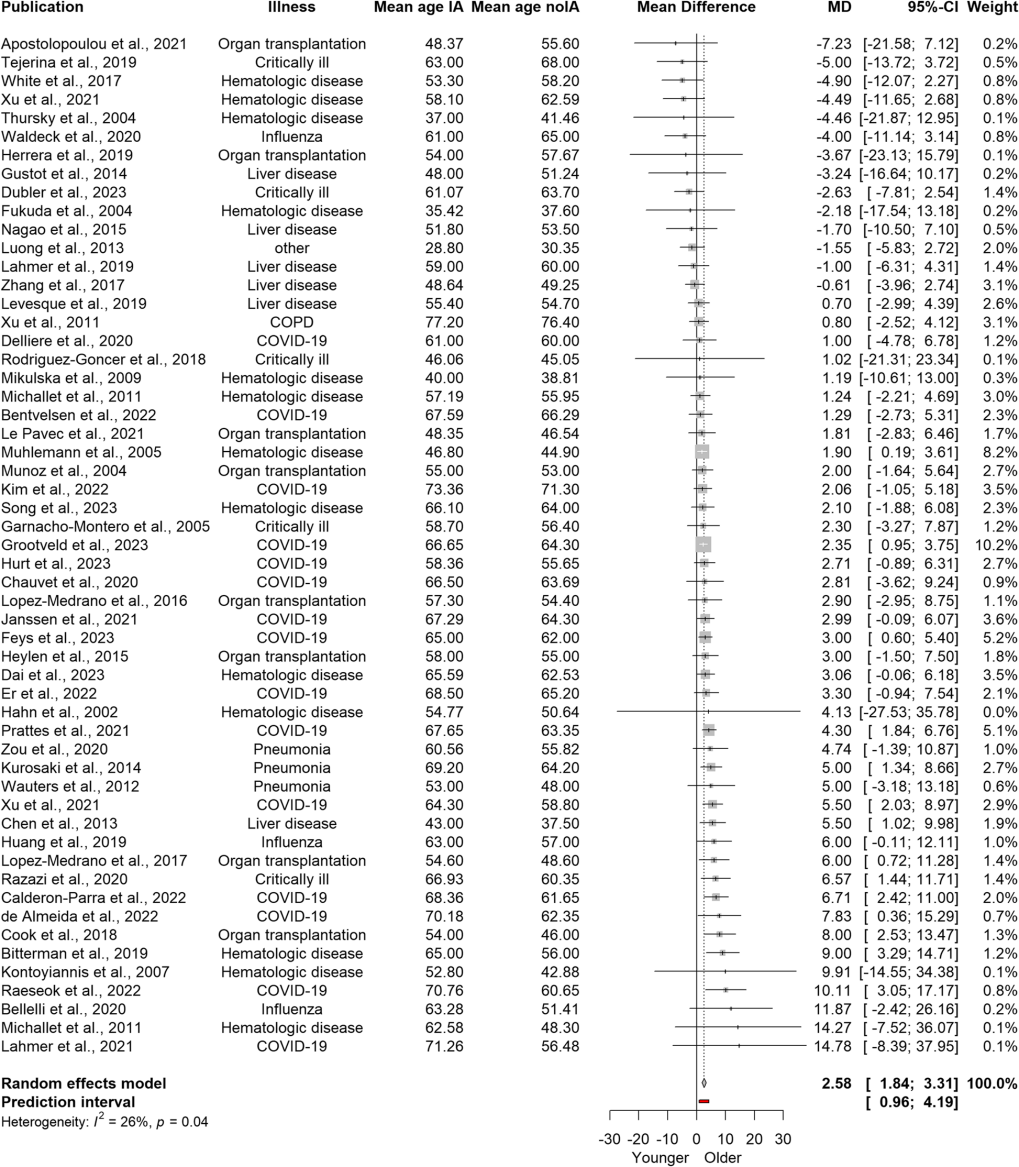
Forest plot illustrating mean age differences in patients with and without invasive aspergillosis

 Evaluation of the difference in age of individuals with and without IA showed that patients who develop IA were generally older (MD = 2.58; 95% CI 1.84–3.31; *p* < 0.0001) (Fig. 3). This means that on average, people who develop IA are approximately two and a half years older than people who do not acquire this disease. The confidence interval suggests that in the universe of comparable studies, the patients with IA can be older by two to three years. Egger’s regression test did not reveal a small study bias (*p* = 0.97, Fig. S3). In addition, a low risk of publication bias was confirmed by examination of the contour-enhanced funnel plot that did not reveal an over-representation of effect sizes in the significant contours (Fig. S4). Also, there was no significant difference (*p* = 0.9685) between studies that reported age as mean (MD = 2.59; 95% CI 1.58–3.60) and studies that initially reported age as median (MD = 2.56; 95% CI 1.29–3.83). Finally, the mean age difference did not differ between cohort (MD = 2.35; 95% CI 1.60–3.10) and case-control (MD = 3.76; 95% CI 2.06–5.46) studies as indicated by the *p*-value of 0.1095.

Our meta-analysis revealed the Q-value of 73.03 with 54 degrees of freedom and a corresponding *p*-value of 0.0432. This allows us to reject the hypothesis that all studies included in this meta-analysis share a common effect size. The I^2^ statistics is 26.1% (95% CI 0.0% − 47.4%), indicating that the variance in observed effects suggests variance in true effects rather than sampling error. The 95% prediction interval, ranging from 0.96 to 4.19 years, suggests that the true mean difference in age varies across populations of patients. This prediction interval indicates that while in certain populations, patients with IA are moderately older than those without this disease, in other populations, the difference in age is trivial.

Therefore, to explore the causes of heterogeneity we implemented meta-regression analyses. The following moderator variables were tested: the year when studies began or ended, the duration of studies, the incidence of IA, data collection before 2014, study design, ICU admission, and antifungal prophylaxis. This test revealed that the variability in the observed effect sizes cannot be accounted for by the included moderators as *p* > 0.05 (Table S4).

**Fig. 4:**
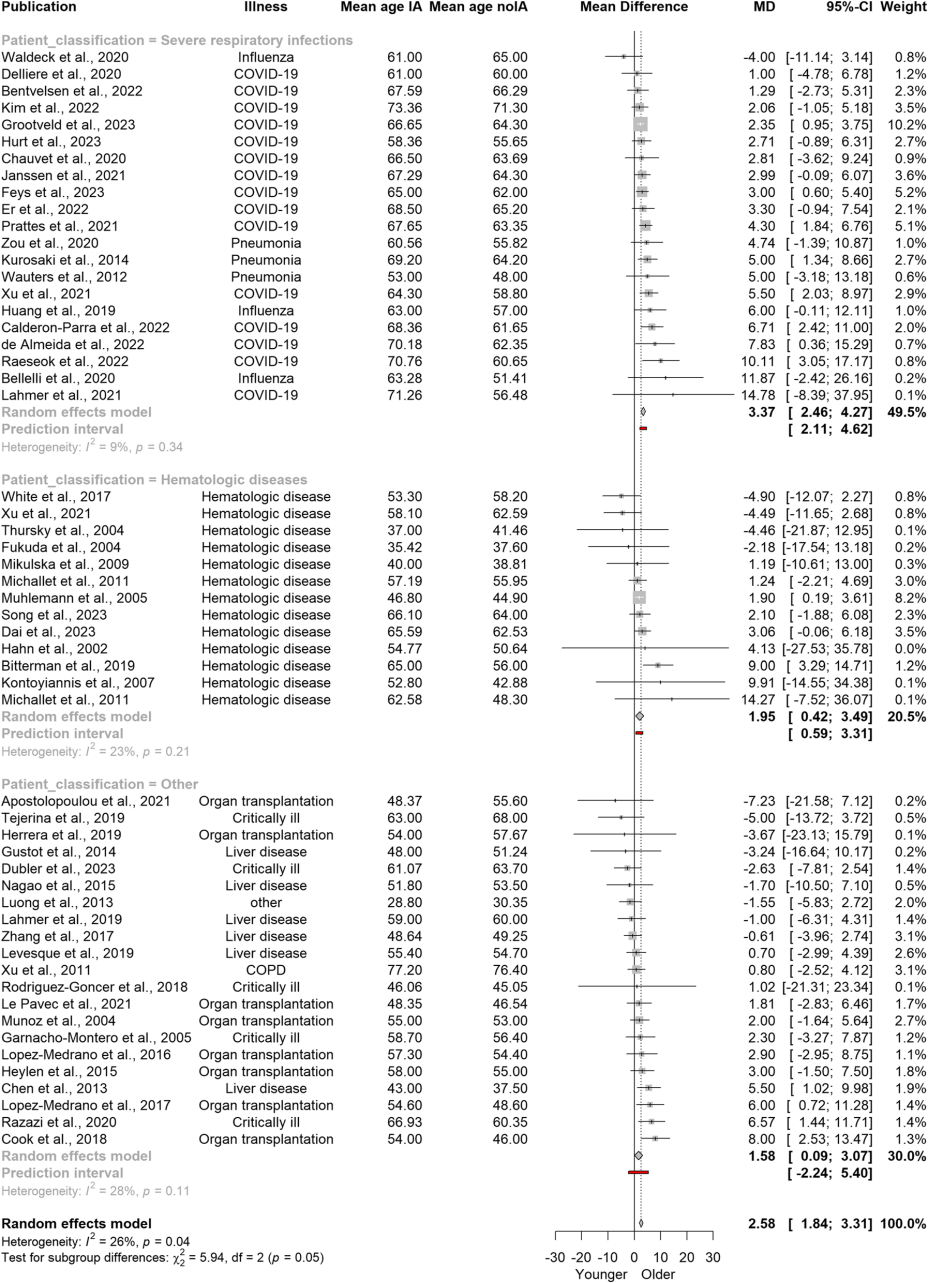
Forest plot illustrating mean age differences in patients with and without invasive aspergillosis stratified into three patient cohorts

 To assess another possible source of heterogeneity we performed a group analysis comparing the age differences in patients with severe pulmonary infections, hematologic patients and all other types of patients (including critically ill individuals, and those with organ disorders and transplantation) unified in one group (Fig. 4). Interestingly, the subgroup analysis revealed that patients with respiratory tract infections were approximately three years older (MD = 3.37) with a 95% CI of 2.46 to 4.27 years. The mean age difference for those with hematologic conditions was lower (MD = 1.95; 95% CI 0.41–3.49). Similarly, patients with other underlying conditions showed a mean age difference of 1.58 years (95% CI 0.09–3.06). The test for subgroup differences indicated that there is a significant difference between these patient classifications (Q = 5.94, df = 2, *p* = 0.0513) and that the mean age difference is likely more substantial in patients with severe lung infections compared to other patient groups.

## Discussion

To our knowledge, this is the first systematic review and meta-analysis study assessing the age of patients with various underlying conditions who developed IA. We found that patients with IA are older than those who do not develop this disease. In total, 13,983 patients from 55 studies were included in the final meta-analysis. On average, patients with IA were found to be about two and a half years older (95% CI 1.84–3.31 years, *p* < 0.0001) than individuals without IA. Therefore, the results of this research suggest that age should be considered while assessing potential risks of developing IA, but additional studies are needed to establish whether age is an independent risk factor and to determine the age at which susceptibility to IA increases.

Our finding is consistent with the previous meta-analyses that evaluated risk factors for IA in a single patient population. In kidney transplantation studies, based on data from six studies, recipient age prior to transplantation was roughly six years (95% CI 3.91–8.01 years) higher for individuals with IA compared to those without IA, where the mean age of patients with IA was over 55 years of age in almost all included studies [94]. Similarly, according to meta-analysis of eight studies, COVID-19 patients diagnosed with IA were typically seven and a half years older (95% CI 2.02–13.03 years) than those who only had COVID-19 [95]. Our study extends the observation in these two cohorts and suggests that such age difference might be true across various populations of patients who are susceptible to *Aspergillus* infections. However, the results of our analysis were heterogenous, suggesting that age difference varies from one patient population to another. In accordance, a narrative review from 2012 reported advanced age as a patient risk factor for IA associated with some but not all underlying conditions including allogeneic hematopoietic stem cells, lung or heart-lung, heart, or small bowel transplantation, leukemia, multiple myeloma, non-Hodgkin’s lymphoma, and burns [3].

Our investigation suggests that the incidence of IA is likely higher in older individuals who already have severe respiratory infections. This could be attributed to the increased possibility of dysregulated immune responses in older individuals. Severe respiratory infections can lead to exacerbated inflammation, resulting in cell infiltration, tissue damage, and hypoxia [96]. As aging is associated with increased basal levels of lung inflammation [97, 98], respiratory infections can be particularly detrimental to aged lungs. Consequently, by causing exaggerated inflammation, primary severe infections create conditions favorable for fungal infection and establishment of the disease [96].

Interestingly, in a previously published study amongst patients with lymphoproliferative diseases and receiving an autologous hematopoietic stem cell transplantation, older age was not observed as a risk factor [99]. However, in another study including patients who underwent bone marrow transplantation, older age was found to be a significant risk factor for the development of late (more than 40 days after transplantation) but not early IA (less than 40 days after transplantation). There, patients, older than 40 were 5-fold times more likely to develop this disease in contrast to younger individuals that were younger than 18 [100]. In our meta-analysis, we grouped all patients with hematologic malignancies together and observed that the age difference between those with IA and those without IA ranged from trivial to moderately older, with a 95% prediction interval of 0.59 to 3.31 years. Such heterogeneity might be explained by patient stratification strategies that were more common in the past. Because elderly people were more predisposed to adverse effects of immunosuppression, younger individuals were more likely to undergo immunosuppressive procedures in preparation and post stem cell transplantation and thus, they were more likely to develop IA in contrast to older hematologic patients. Therefore, the underrepresentation of elderly individuals in the “hematology” subgroup would explain such heterogeneity of age difference.

The age difference within the group comprising critically ill patients, individuals with organ disorders, and patients undergoing organ transplantation showed considerable heterogeneity. Notably, the wide prediction interval of -2.24 to 5.40 years indicates that, in these cohorts, while some individuals with IA were slightly younger than their counterparts, others could be substantially older. To gain a deeper understanding of these variations and their clinical implications, future research should investigate the specific factors or conditions that contribute to such heterogeneity.

Overall, our findings suggest that older age may put patients at additional risk of developing IA. This has an important clinical significance because advanced age might also be associated with an increased mortality rate if the disease is established. For example, older age was an independent risk factor for mortality among patients with IA who are critically ill [101] or suffer from acute myeloid leukemia [30]. An 11-year follow-up report study from Taiwan has found that in-hospital mortality from IA increased with age being the highest for the 80 + age group [102]. However, this requires further investigation. In another study, where only ICU patients with IA were included, mortality was not different for individuals older or younger than 75 years old [103].

The main strength of this study is its large sample size of participants: the control group consisted of 12,045 patients, while the IA group included 1,938 individuals. This contrasts with a typical clinical study of patients with IA that on average includes only 250 and 30 participants in each group respectively. Other strengths include the pre-registered protocol basis (study design and analysis plan created prior to analysis), and the application of a random-effects model to account for substantial heterogeneity among included studies. Another strength is the inclusion of data from patients with a wide range of underlying conditions and from different countries that allow us to synthesize a data set representative of real-world populations affected by IA. Our present study also had several limitations: lack of correction for important confounders such as chronic conditions, gender, or environmental factors. In addition, some studies did not distinguish between probable, proven, or putative IA diagnosis and thus could introduce false positive results. Selection bias may exist because we included only papers with full reports in English.

Another limitation of the study includes a limited direct utility for decision-making parties. Although we suggest a potential association between older age and increased frequency of IA, we were unable to provide specific age constraints. Future cross-sectional studies should aim to identify age thresholds at which susceptibility to IA is heightened [104, 105]. Additionally, to perform regression analysis on IA incidence and age while accounting for confounding variables, individual patient data would be necessary.

## Conclusion

To summarize, our systematic review and meta-analysis found that patients with IA are older than those without the disease. This finding warrants further research into determining whether older age is an independent risk factor for the disease. Our study extends previous observations and suggests that the age difference may be true across various patient populations susceptible to IA. Advanced age may also be associated with increased mortality rates among patients with IA, which underscores the importance of optimized risk stratification strategies. While our study had several limitations, it represents a significant step toward understanding the relationship between age and IA development.

### Supplementary Information

**Additional file 1:** **Supplemental Table 1.** PRISMA checklist that provides locations where each item of systematic review and meta-analysis is reported. **Supplemental Table 2.** Meta-analysis results before and after outliers (determined by sensitivity analysis) were removed. **Supplemental Table 3.** Newcastle - Ottawa assessment of non-randomized studies included in meta-analysis. **Supplemental Table 4.** Meta-regression analysis of age difference in years. **Supplemental Figure 1.** Baujat plot illustrating contribution of individual studies to the overall heterogeneity. **Supplemental Figure 2. **Leave-One-Out meta-analysis illustrating forest plots, where pooled effects were recalculated with one study omitted each time. **Supplemental Figure 3.** Funnel plot depicting the small-study effect. **Supplemental Figure 4.** Contour-enhanced funnel plot with colors representing the significance level of each individual studies.

## Data Availability

The datasets generated and analysed during the current study are available in the Open Science Framework repository, [https://osf.io/69e8u/?view_only=c700416f336c41cbbee8627c0492ac77].
