# Supplementary material for: Age difference of patients with and without invasive aspergillosis: a systematic review and meta-analysis
Source: BMC Infect Dis. 2024 Feb 19;24:220. doi: 10.1186/s12879-024-09109-2 (PMC10875810; doi:10.1186/s12879-024-09109-2)

**Supplemental materials**

Age difference of patients with and without invasive aspergillosis: A systematic review and meta-analysis

Elena Shekhova*^1^, Fabián Salazar^1^, Alessandra Da-Silva Dantas^2^, Tanmoy Chakraborty^1^, Eva L Wooding^1,3^, P Lewis White^4,5^ and Adilia Warris^1^

^1^Medical Research Council Centre for Medical Mycology at the University of Exeter, Geoffrey Pope Building, University of Exeter, Stocker Road, EX4 4QD, Exeter, UK

^2^ School of Dental Sciences, Newcastle University, Newcastle upon Tyne, Framlington Place, NE2 4BW, Newcastle upon Tyne, UK

^3^ Royal Devon and Exeter Hospital, Exeter EX2 5DW, UK

^4^Public Health Wales Microbiology Cardiff, Cardiff University, UHW, Cardiff, UK

^5^Centre for Trials Research/Division of Infection and Immunity, Cardiff University, UHW, Cardiff, UK

*Corresponding Author:

Elena Shekhova

E: [shekhova.elena@yandex.com](mailto:shekhova.elena@yandex.com)


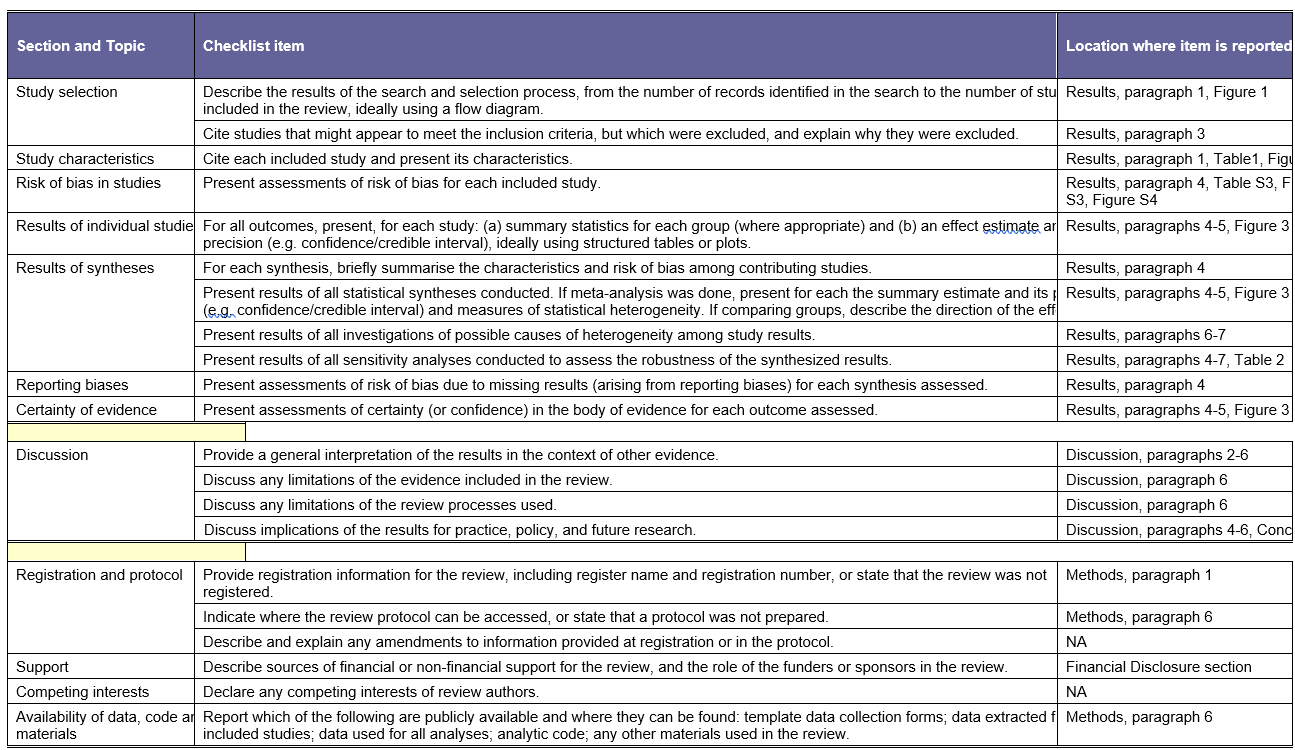

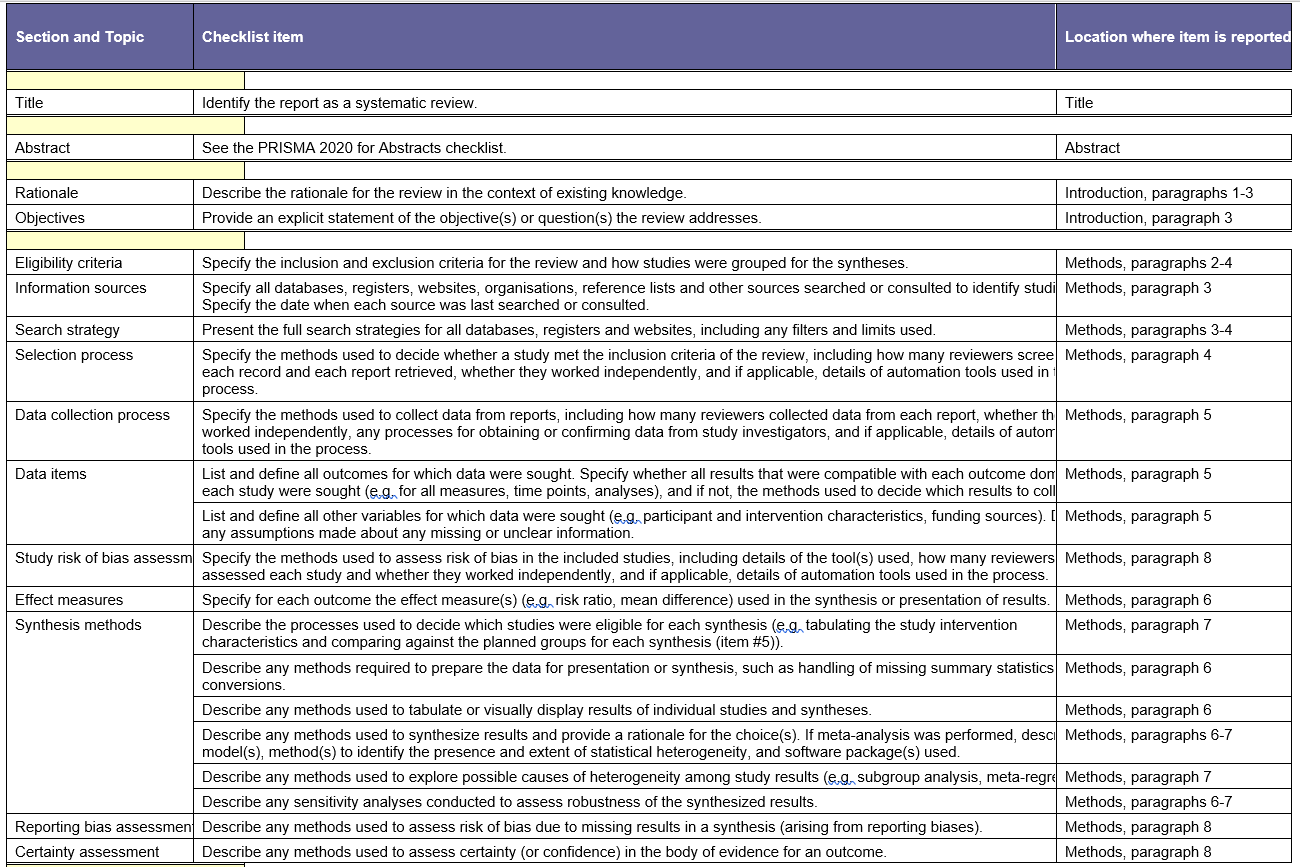
**Supplemental Table 1**. PRISMA checklist that provides locations where each item of systematic review and meta-analysis is reported

| Analysis | MD | 95%CI | *p* | 95% PI | I2 | 95% CI |
| --- | --- | --- | --- | --- | --- | --- |
| With outliers | 2.4243 | 1.1846-3.6640 | 0.0002 | -5.4362- 10.2848 | 68.7% | 59.6%- 75.7% |
| Outliers removed | 2.5777 | 1.8439-3.3115 | <0.0001 | 0.9624-  4.1930 | 26.1% | 0.0%- 47.4% |

**Supplemental Table 2**. Meta-analysis results before and after outliers (determined by sensitivity analysis) were removed

Removed outliers: Waldeck et al., 2023", "Chao et al., 2021", "Katada et al., 2022", "Gu et al., 2021", "Chen et al., 2020", "Seok et al., 2020", "Sharma et al., 2020", "Napolioni et al., 2019", "Kaya et al., 2017", "Schwarzinger et al., 2013"

**Supplemental Table 3**. Newcastle - Ottawa assessment of non-randomized studies included in meta-analysis

AHRQ - Agency for Healthcare Research and Quality

**Supplemental Table 4.** Meta-regression analysis of age difference in years.

|  | | | | | | |
| --- | --- | --- | --- | --- | --- | --- |
| Variable | Coefficient | Standard error | Degrees of freedom | p value | I^2, % | R^2, % |
| Year end of study | 0.0775 | 0.0493 | 52 | 0.1220 | 9.46 | 0.00 |
| Year start of study | 0.0381 | 0.0382 | 52 | 0.3238 | 9.32 | 0.00 |
| Duration of study | 0.0280 | 0.0753 | 52 | 0.7111 | 12.33 | 0.00 |
| Incidence of IA | 0.0022 | 0.0379 | 37 | 0.9542 | 13.45 | 0.00 |
| Studies ended before 2014 | -0.8130 | 0.7772 | 52 | 0.3004 | 8.69 | 4.03 |
| Study design | -1.3132 | 0.9192 | 53 | 0.1590 | 16.47 | 0.00 |
| ICU admission | 0.2626 | 0.7595 | 53 | 0.7309 | 12.61 | 0.00 |
| AF prophylaxis | -1.9707 | 0.9906 | 53 | 0.0518 | 10.77 | 0.00 |
| Note: I^2 - residual heterogeneity / unaccounted variability, R^2 - amount of heterogeneity accounted for, AF - antifungals | | | | | | |

**Supplemental Figure 1**. Baujat plot illustrating contribution of individual studies to the overall heterogeneity


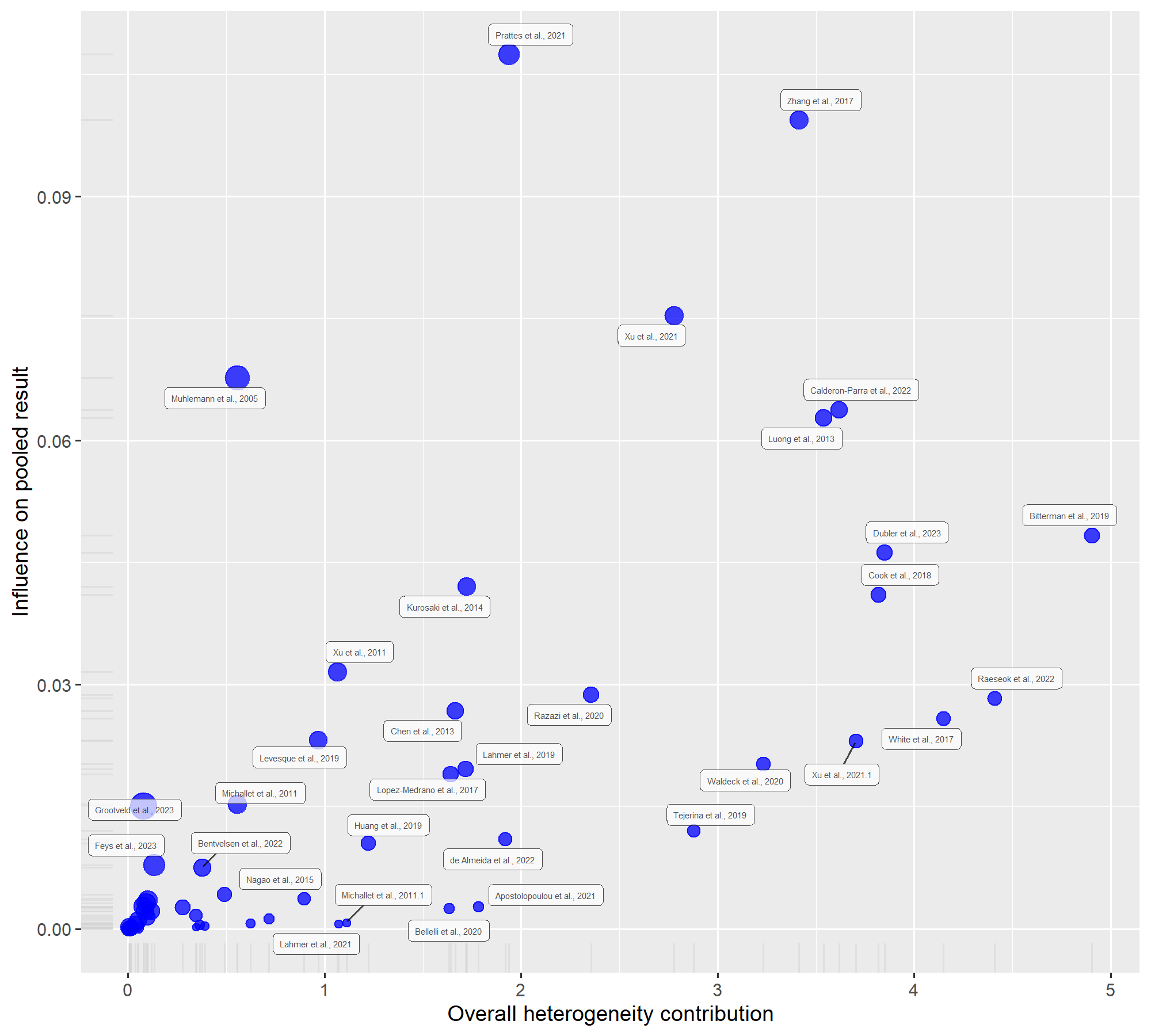


**Supplemental Figure 2**. Leave-One-Out meta-analysis illustrating forest plots, where pooled effects were recalculated with one study omitted each time


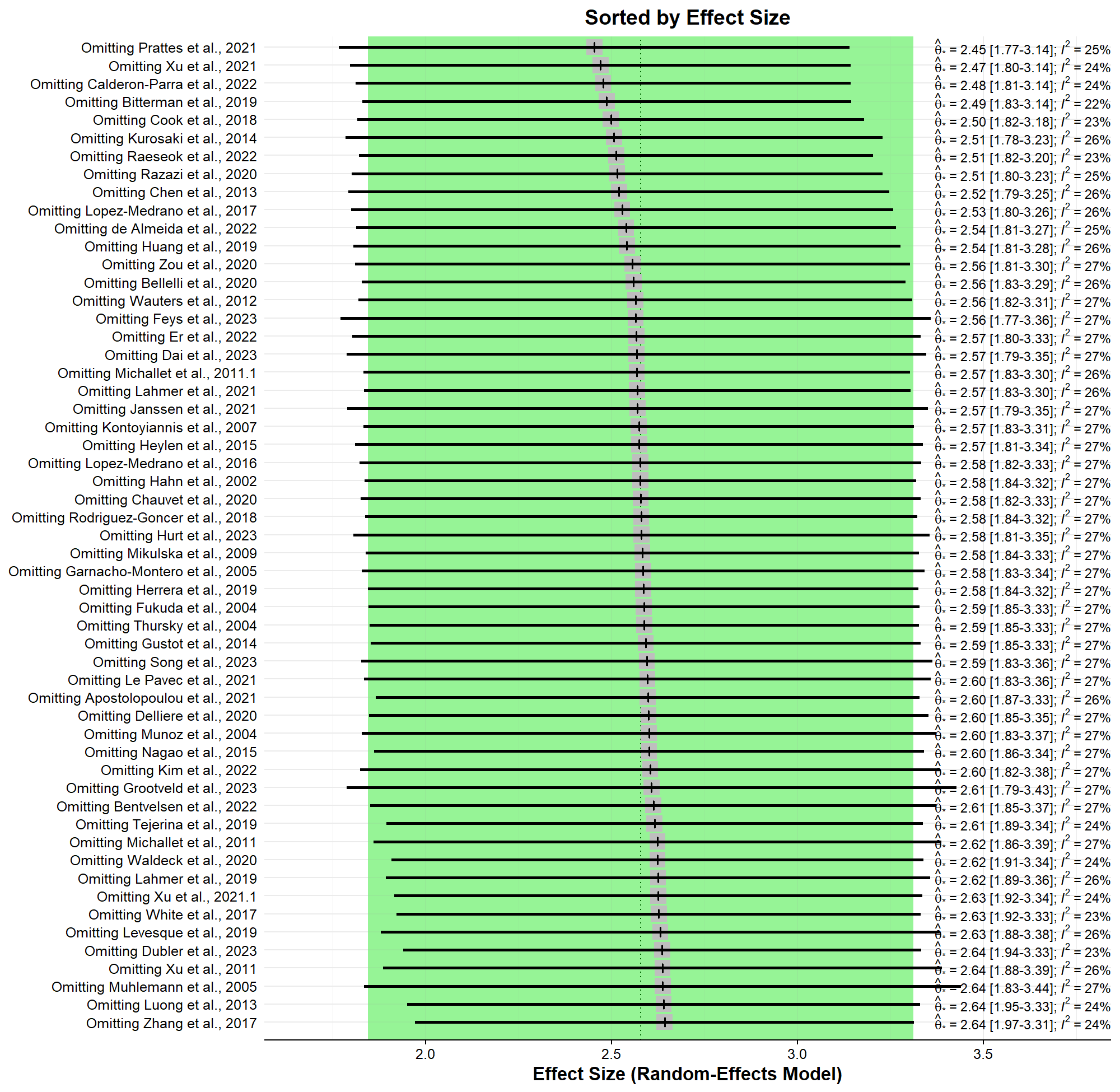


**
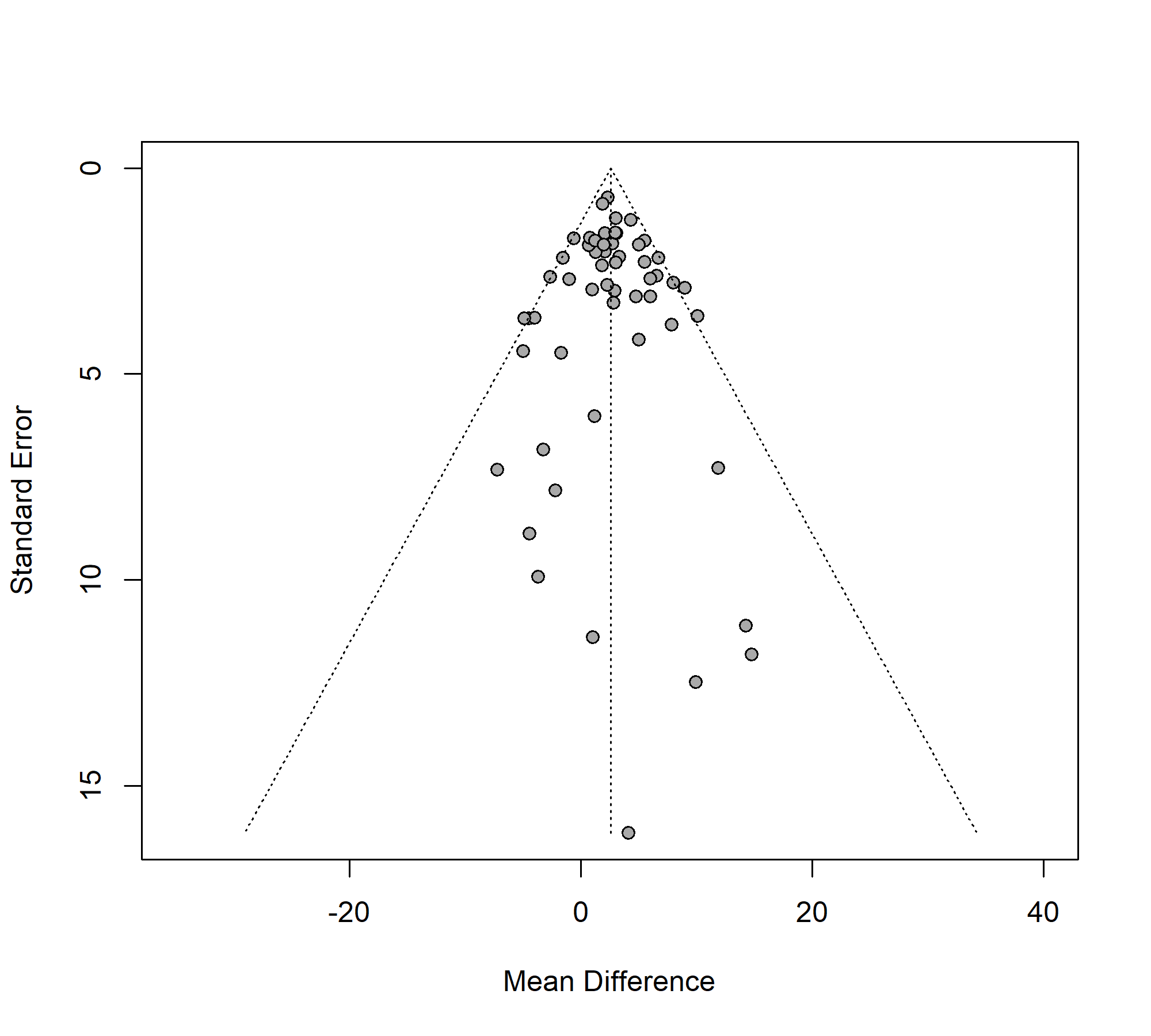
Supplemental Figure 3**. Funnel plot depicting the small-study effect

**Supplemental Figure 4**. Contour-enhanced funnel plot with colors representing the significance level of each individual studies


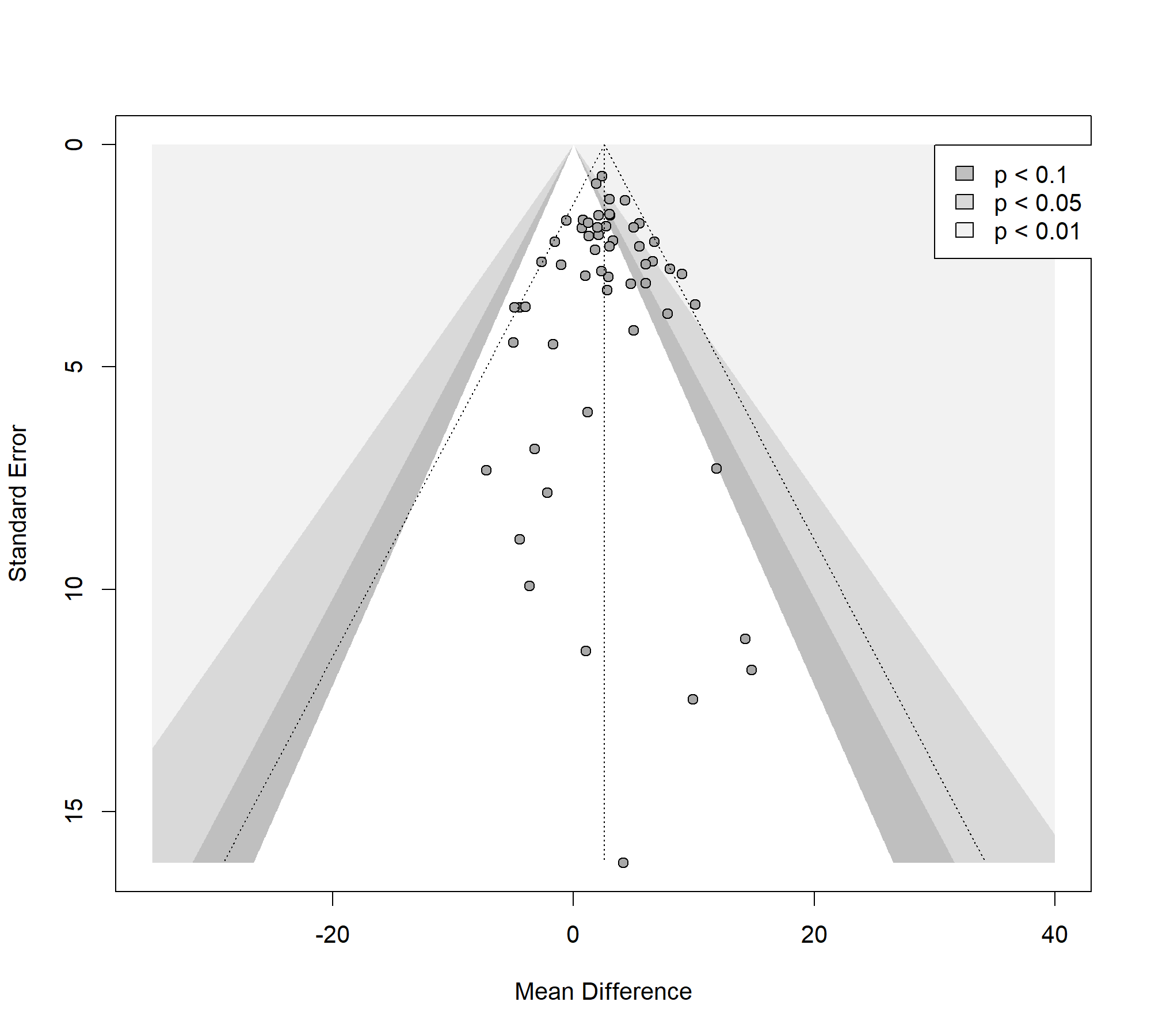

Supplement: Supplementary file 1 — Additional file 1: Supplemental Table 1. PRISMA checklist that provides locations where each item of systematic review and meta-analysis is reported. Supplemental Table 2. Meta-analysis results before and after outliers (determined by sensitivity analysis) were removed. Supplemental Table 3. Newcastle - Ottawa assessment of non-randomized studies included in meta-analysis. Supplemental Table 4. Meta-regression analysis of age difference in years. Supplemental Figure 1. Baujat plot illustrating contribution of individual studies to the overall heterogeneity. Supplemental Figure 2. Leave-One-Out meta-analysis illustrating forest plots, where pooled effects were recalculated with one study omitted each time. Supplemental Figure 3. Funnel plot depicting the small-study effect. Supplemental Figure 4. Contour-enhanced funnel plot with colors representing the significance level of each individual studies. [file 12879_2024_9109_MOESM1_ESM.docx]
